# The Evolution of Residual Stress in Rib-Diaphragm Joints of Orthotropic Steel Decks Subjected to Thermal Cutting and Welding

**DOI:** 10.3390/ma13173804

**Published:** 2020-08-28

**Authors:** Yongming Xiong, Chuanxi Li, Zhuoyi Chen, Jun He, Haohui Xin

**Affiliations:** 1 Key Laboratory of Bridge Engineering Safety Control by Department of Education, Changsha University of Science and Technology, Changsha 410114, China; xiongyming96@gmail.com (Y.X.); lichx@csust.edu.cn (C.L.); chenzhuoyi@csust.edu.cn (Z.C.); 2 School of Civil Engineering, Changsha University of Science and Technology, Changsha 410114, China; 3 Civil Engineering and Geosciences, Delft University and Technology, 2600 AA Delft, The Netherlands

**Keywords:** orthotropic steel deck (OSD), rib-diaphragm joint, residual stress, thermal-mechanical analysis, thermal cutting, welding

## Abstract

Residual stresses change the stress ratio of fluctuating stresses, hence seriously affect the fatigue life of orthotropic steel decks (OSDs) under traffic loading. Residual stress distributions near the U rib-diaphragm joints are very complicated and need to be investigated further. In this paper, a systematic method has been proposed for calculating the residual stress field in the joint of U rib and diaphragm due to thermal cutting and welding. Firstly, a mathematical model of cutting heat sources was established to predict the temperature field. Then, a numerical elastoplastic thermomechanical model was built to predict the residual stress evolutions in a diaphragm-rib joint through the whole fabrication process involving flame cutting and welding. Moreover, the simulated temperature contours at the fusion zone and the residual stress distributions in the rib-diaphragm joint were compared and verified against the experimental ones. The numerical results showed a great agreement with the experimental ones, indicating that the heat source model can be used to accurately predict the temperature field during flame cutting. Finally, the validated numerical model was utilized to conduct parametrical analyses on the effects of thermal processing rates, e.g., the cutting and welding speeds and on the residual stress distribution in the rib-diaphragm joint. The results indicate that a faster cutting speed and a slower welding speed can decrease the residual stress magnitude at the rib-diaphragm joints and reduce the high-stress zone near the diaphragm cutouts.

## 1. Introduction

Orthotropic steel decks (OSDs) are widely used in long-span bridges because of their superior features, such as overall light self-weight, fast construction, high loading capacity, etc. [1,2]. However, OSDs are prone to fatigue problems under repeated traffic loads [3]. Such fatigue damages have been observed after only about a decade of service in the Rio-Niterói Bridge [4], the Jiangyin Yangtze River Bridge [5], and the Guangzhou Pingsheng Bridge [6]. Furthermore, the fatigue disease of the Severn bridge has been classified by Cuninghame et al. [7], in which the U rib-diaphragm joints are prone to fatigue damage. The Japanese Steel Structure Committee also found that the number of fatigue cracks at these locations accounts for 56.3% of the total number of fatigue cracks in OSDs [8].

At present, the research on the fatigue behavior of OSDs has achieved academic achievements on the aspects of wheel loads, weld joints, initial imperfections, and stress distributions. The approaches of damage tolerance design and safe life design based on fracture mechanics are widely applied in design standards of steel structure [9,10]. The S-N curves of the joints are often obtained through a large number of experiments; experimental results indicate that the welding residual stress near the weld joints has a great influence on the fatigue life [11,12]. Therefore, previous research has paid more attention to the residual stress influences on welded joints [13,14,15]. The residual stresses in OSDs were analyzed by finite element method, and finite element analysis (FEA) results revealed that the variation of welding parameters has a significant influence on residual stress distributions [16,17]. The fatigue life of welded joints in OSDs can be evaluated more accurately in consideration of measured traffic flow data and residual stress distributions in welded joints [18,19,20]. From the above-mentioned works, most scholars have focused on the rib-deck joints [21], rather than the U rib-diaphragm joints. Just a few publications analyzed the welding residual stress in the joints of diaphragm and U rib [22]. However, the manufacture of U rib-diaphragm joints includes many processes and the residual stress distribution can be caused not only by welding, but also by flame cutting. Previous studies showed that the joints between the diaphragms and U ribs of steel box girders suffer compressed stress under the static load of vehicles [23], which are theoretically unlikely to generate cracks. So, this problem of fatigue has increasingly become one that plagues scholars around the world. In addition, the magnitude of residual stress sometimes can even be more than the yield strength of the steel [24,25,26], the stress on the U rib-diaphragm joints might be changed from compression to tension considering the effect of residual stress under repeated loading of vehicles [27,28,29], that may explain the occurrences of fatigue cracks at cutout of U rib-diaphragm joints.

Nonuniform temperature distributions and local plastic deformations inevitably result in complex residual stress fields in OSDs during manufacturing [30]. The manufacturing process of OSDs includes dividing the steel plates into designed shapes by thermal cutting and assembling them into OSDs by welding—these high temperature processing steps result in a significant effect on residual stresses [31]. However, existing literature lacks quantitative data on the changes in residual stress field induced by the continuous process of flame cutting and welding. The residual stress field of rib-diaphragm joints cannot be simply defined as a superimposition of the residual stress caused by cutting and by welding. The evolution of residual stresses considering the continuous process of flame cutting and fillet welding should be investigated to understand fatigue mechanisms of rib-diaphragm joints in OSDs.

In the present study, a thermo-mechanical method has been proposed to predict the residual stress distribution in the joint between U rib and diaphragm during the thermal cutting and welding process. Firstly, a simplified finite element model for cutting heat sources was proposed to predict the temperature field of the joint due to cutting and welding. Then, the numerical models of a diaphragm-rib joint considering thermo-mechanical coupling was established by finite element (FE) software ABAQUS, for investigating the stress distribution of cutout in the diaphragm caused by oxy-ethylene flame cutting, as well the residual stress to diaphragm-rib joints induced by fillet welding. Moreover, the residual stress distributions were measured by two methods, including the x-ray diffraction method and the hole-drilling (HD) method. The measurement results were compared with simulation ones, to verify the proposed FE model. Finally, parametrical analyses were conducted using validated numerical models to investigate the effect of thermal processing rates, e.g., the cutting and welding speeds, on the residual stress distribution in rib-diaphragm joints. The present investigation may provide some insights into the thermal residual stress distribution of diaphragm-rib joints in OSDs.

## 2. Numerical Simulation Model

### 2.1. Geometry and Method

Numerical analysis was performed by finite element (FE) software ABAQUS (ver. 6.4.1, Hibbitt, Karlsson & Sorensen, Inc., Johnston, NC, USA) [32]. According to the fabrication processes of U rib-diaphragm joints in OSDs, the distribution of residual stress was analyzed through a two-step thermo-mechanical analyses using the FE method: (1) a rectangular steel plate was cut into the diaphragms by thermal cutting; (2) the diaphragm was welded to the U rib and top plate, which are the other two parts of an OSD.

**Figure 1:**
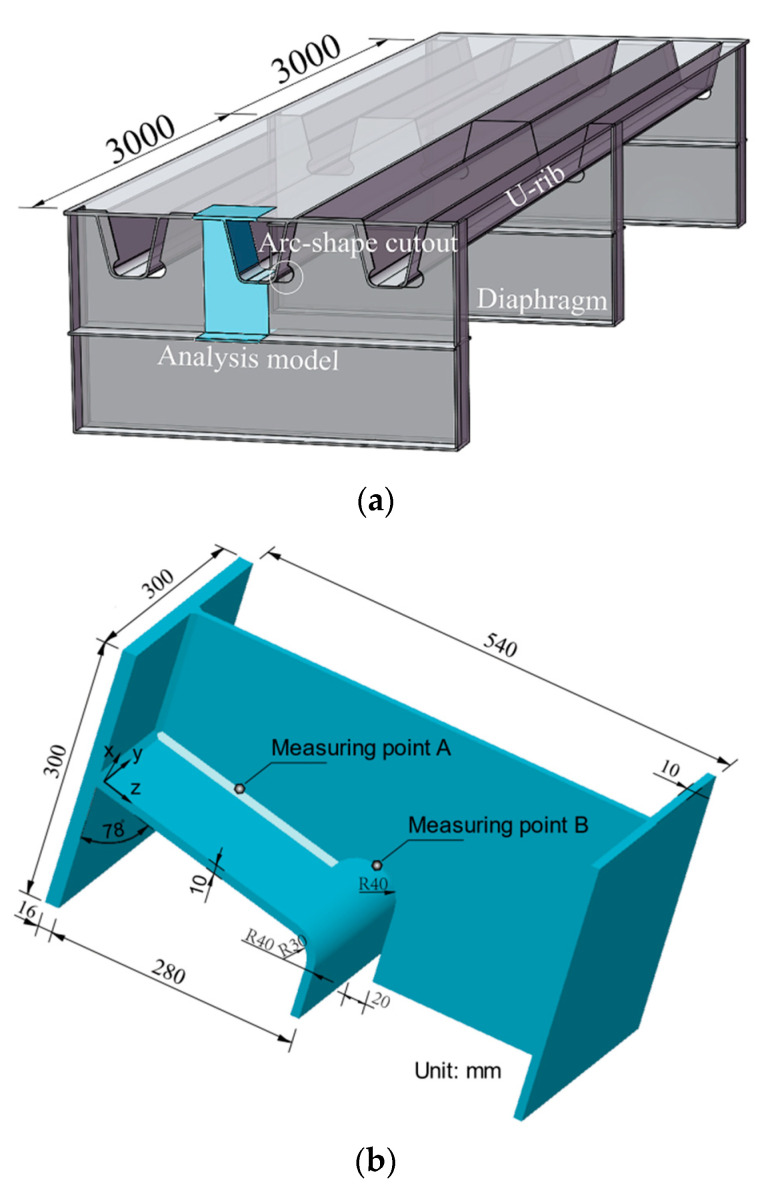
A typical U rib-diaphragm joint in OSD (unit: mm): (**a**) orthotropic steel bridge deck; (**b**) half symmetrical model of a U rib-diaphragm joint.

In order to analyze the characteristics of the residual stress distribution on the free edge of the diaphragm, a typical U rib-diaphragm joint in OSD was selected as the research object, as shown in Figure 1. Only half of the joint was built considering the symmetric geometry, as shown in Figure 1b.

From Figure 1, a 300 × 540 mm rectangular plate with thickness of 10 mm was established as the model of the diaphragm with a circular cutout (radius of 40 mm) for the cutting process. After calculating the residual stress distributions caused by the flame cutting of the diaphragm, a finite element model (FEM) with initial cutting residual stress in the diaphragm was established to analyze the distributions of residual stress in the rib-to-diaphragm welded joints. A transverse stiffener (300 × 10 mm) was also added at the diaphragm to simulate the confinement effect on the diaphragm as shown in Figure 1b. The details of the U rib–diaphragm are depicted in Figure 1b, in which the U rib has a height of 280 mm, a width of 180 mm at bottom plate, and a thickness of 10 mm, a segment of the stiffened top plate (300 × 300 × 16 mm) in the OSD was selected. Fillet welding was used to weld the diaphragm to the top steel plate and U rib, and the size of fillet welding was 6.5 mm.

The simulation of a U rib-diaphragm joint during the fabrication process was divided into three stages (the numbers in parenthesis indicate the time durations of each stage): (1) a plate was cut into the diaphragm of the OSD (0 s–1.09 s); (2) welding the diaphragm to the top steel plate (561.09 s–634.06 s); and (3) welding the diaphragm to the U rib (1034.06 s–1145.57 s). At the completion of each stage, the component was cooled to 20 °C before proceeding the next stage (i.e., the model was cooled to an ambient temperature of 20 °C during the time intervals of 61.09 s–561.09 s, 634.06 s–1034.06 s, and 1145.57 s–1645.57 s).

### 2.2. Material Properties

**Figure 2:**
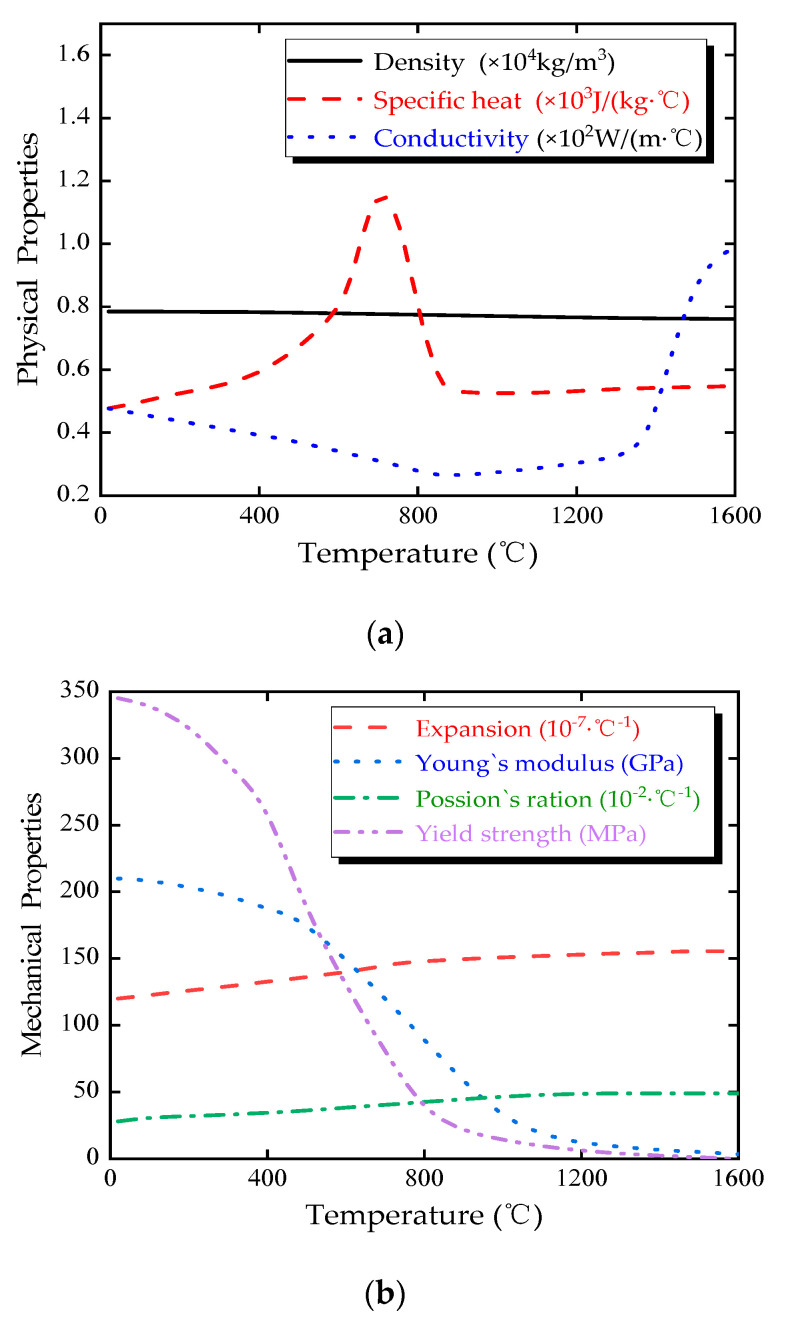
Temperature-dependent properties of Q345 Steel: (**a**) physical properties; (**b**) mechanical properties.

For the numerical simulations, the hot-rolled low alloy steel Q345 with a nominal yield strength of 345 MPa was used for the OSD. The temperature-dependent mechanical and physical properties of Q345 steel have been obtained from some references [12,33,34], which are presented in Figure 2.

### 2.3. Boundary Conditions

**Figure 3:**
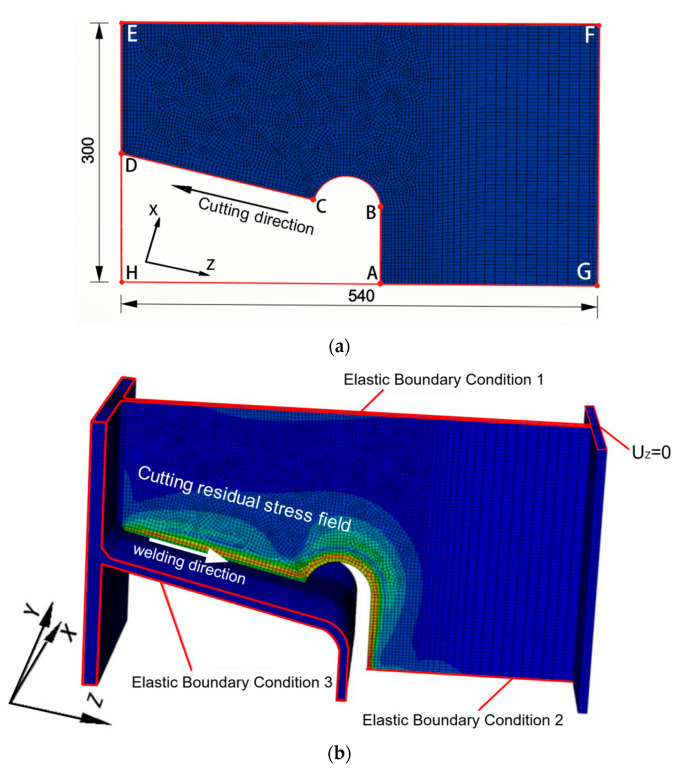
Finite element meshes and boundary conditions: (**a**) schematic diagram of cutting; (**b**) schematic diagram of welding.

A plate with the same dimension as the diaphragm in Figure 1b was established as a cutting model (Figure 3a), where the predefined cutting slit width is 2 mm. After a sensitivity analysis of mesh sizes, a mesh size of 2 mm was chosen to divide the solid element (C3D8T) for cutting the rectangular plate. In order to reduce computational time, the element size was only refined near the heat-affected zone, and the total number of elements for the model was 48,966. Figure 3a shows the coordinate system for the cutting model with the following boundary conditions: (1) the edges AG and EF were set as symmetrical constraints; (2) the edge FG was set as the normal displacement constraint; and (3) the edge ED was assigned as the free edge. The cutting began at point A, then passed through points B and C, and finally reached point D at completion.

The FE model for the analysis of the welding process is shown in Figure 3b, which consists of a U-rib, a diaphragm with bottom stiffener, and a top steel plate, and was composed of a total of 110,872 solid elements (C3D8T). Since boundary conditions have a great influence on the distribution of residual stress, strong constraints might be unfavorable for structural safety [35]. Hence, an elastic boundary condition, based on continuum elastic theory, was selected considering the ductility and flexibility of the U rib and top steel plate, as well the constrain effect of transverse diaphragm stiffeners, on the joint model (Figure 3b).

## 3. Heat Source Model and Thermal Analysis

During the process of oxy-ethylene flame cutting, the high temperature flame will generate heat flux on the surface of the plate—the effect of the extremely high flame temperature is dominant at the upper surface of the cutting domain [36]. The temperature distribution in the lower part of the plate near the cutting line was assumed to be similar to that at the middle region along the direction of plate thickness [37].

**Figure 4:**
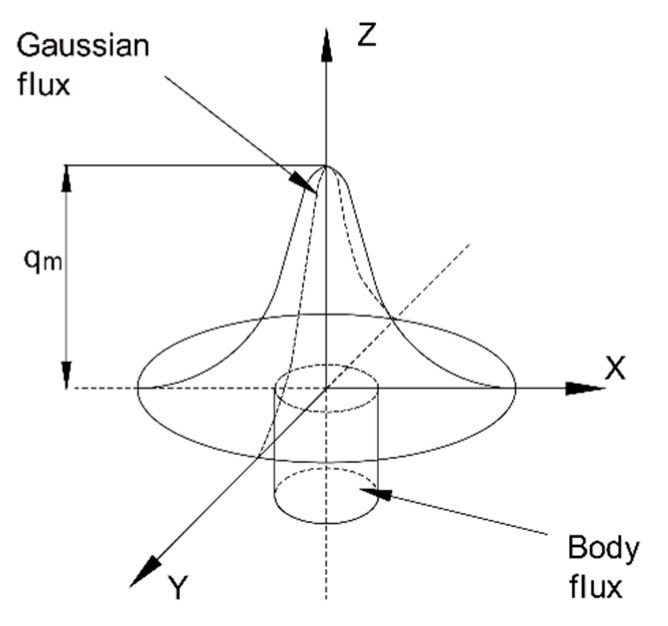
Schematic diagram of the cutting heat source.

Thiebaud et al. [33] and Bae et al. [38,39] studied the temperature history indicators of two different thick steel plates respectively and analyzed that temperature changes along the cutting line. The corresponding temperature distribution in the heat-affected zone (HAZ) was also analyzed in detail using validated heat source parameters. Chen et al. [40] conducted extensive experimental research on the flame cutting of 10 mm-thick Q345 steel plates, and determined the average width of the HAZ to be 0.75 mm. Based on the previously proposed theories of heat sources in welding from above literatures [33,34,38,39], the following heat source model by cutting was applied, as shown in Figure 4. In addition, three assumptions were adopted in the model:

a. the heat flux was considered as a load, and all thermal properties were expressed as a function of temperature.
b. the cutting flame acting on the plate surface was expressed by heat flux in terms of Gaussian functions.
c. the heat generation of the droplets can be simplified, and the heat from the chemical reaction at the cutting line was assumed to be uniformly distributed.

As shown in Figure 4, the heat distribution induced by the cutting flame is assumed to be a Gaussian distribution on the surface of cutting area (i.e., the x-y plane), and the combustion heat of steel plate is uniformly distributed in a cylindrical volume. The corresponding equations (Equations (1) and (2)) of heat source are obtained as follows:

Gaussian heat source on the surface:

(1) $$\begin{array}{cc} q_{g\left(x,y,z\right)}=\frac{3\eta Q_{1}}{\pi R_{1}^{2}}exp\left(\frac{-3\left(x^{2}+y^{2}\right)}{R_{1}^{2}}\right) & \begin{array}{cc} z=0 & \end{array}\; \end{array}$$

**Figure 5:**
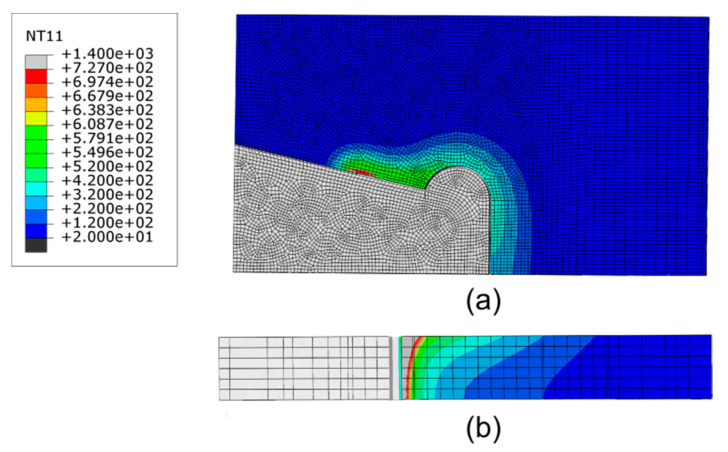
Temperature (unit: °C) contours in the fusion zone and heat-affected zone (HAZ) of the cutting domain: (**a**) top of the cutting line and (**b**) cross-section of the cutting line.

Uniform distribution of heat in a cylindrical volume:

(2) $$\begin{array}{cc} q_{c\left(x,y,z\right)}=\frac{Q_{2}}{\pi HR_{2}^{2}} & \begin{array}{cc} x^{2}+y^{2}\leq R_{2}^{2} & \end{array} \end{array}$$

where, the $q_{g\left(x,y,z\right)}$ and $q_{c\left(x,y,z\right)}$ denote the gaussian and cylindrical heat sources respectively. *R*_1_ is the effective radius of the superficial gaussian heat flux, the heat efficiency η is set to be 0.3, and *Q*_1_ is the combustion heat at surface in Equation (1); *R*_2_ is the effective radius of chemical reaction zone of steel with oxygen, *Q*_2_ is the oxidation energy of steel, and *H* is the effective height of cylindrical distribution in Equation (2). In the present study, *H* was taken as the thickness of the steel plate, and *R*_2_ was identical to the half width of the cutting seam. In addition, the comparison of simulated and experimental cross-section profiles is considered as a standard for judging the accuracy of numerical simulation [34]. So, the isotherms (Figure 5) were adjusted in accordance with the experimental cross-section [33,36,37,38,39,40], which was considered to satisfy the accuracy of the study. The total parameters for the cutting heat source model are summarized in Table 1.

The temperature distribution was estimated by numerical simulations with the proposed model of the heat source when the cutting torch passed over the center of the solution domain, as shown in Figure 5. The temperature at the cutting line was defined to be the melting temperature of the steel plate. The eutectoid phase transition occurred when the temperature of the steel exceeded the Ac1 temperature line (727 °C)—the material properties of steel change significantly, which can be used to identify the HAZ of the steel plate [40]. In Figure 5b, the influenced area at the upper of the solution domain is wider than that at lower because of the direct action of the flame on the upper surface. At the lower part near the cutting line, the temperature distribution is similar to that in the central region along the direction of plate thickness.

**Figure 6:**
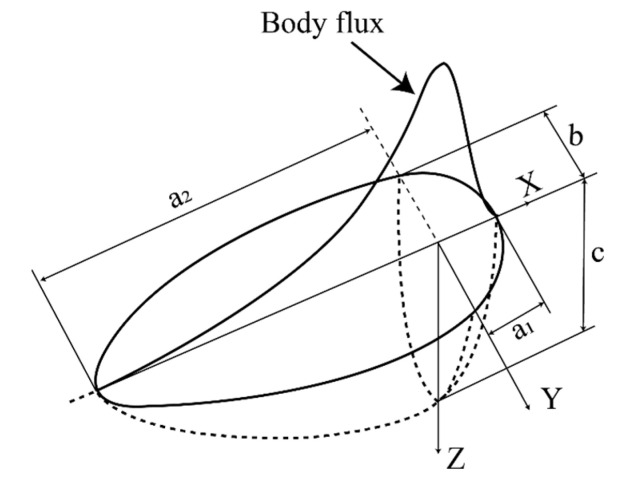
Schematic diagram of welding heat source.

During the assembling process of the OSD, the diaphragm was assembled to the top steel plate and U rib by double-sided fillet welding. The welding heat source was simulated as a moving heat flux over the cross-section of the specimen using a double ellipsoidal distribution model [41,42], as shown in Figure 6.

The front and rear portions of the heat source are represented by the following equations:

For the front quadrant heat flux:

(3) $$q_{f}(x,y,z)=\frac{6\sqrt{3}f_{f}Q}{a_{f}bc\pi \sqrt{\pi }}\exp(-\frac{3x^{2}}{a_{f}{}^{2}}-\frac{3y^{2}}{b^{2}}-\frac{3z^{2}}{c^{2}})$$

For the rear quadrant heat flux:

(4) $$q_{r}(x,y,z)=\frac{6\sqrt{3}f_{r}Q}{a_{r}bc\pi \sqrt{\pi }}\exp(-\frac{3x^{2}}{a_{r}{}^{2}}-\frac{3y^{2}}{b^{2}}-\frac{3z^{2}}{c^{2}})$$

where, the total input power is Q=ηIU, the arc efficiency  η is set to be 0.9 and $f_{f}\;\mathrm{and}\;f_{r}\;\left(f_{f}+f_{r}=2\right)$ are the heat distribution functions of the front and the rear parts in ellipsoid, respectively. The a, b, and c are the longitudinal-width, transverse-width, and depth of the ellipsoid (unit: mm), respectively. Based on the recommendations by Wang et al. [22] and Goldak et al. [42], the values of these parameters used for calibrating the heat source are given in Table 2.

**Figure 7:**
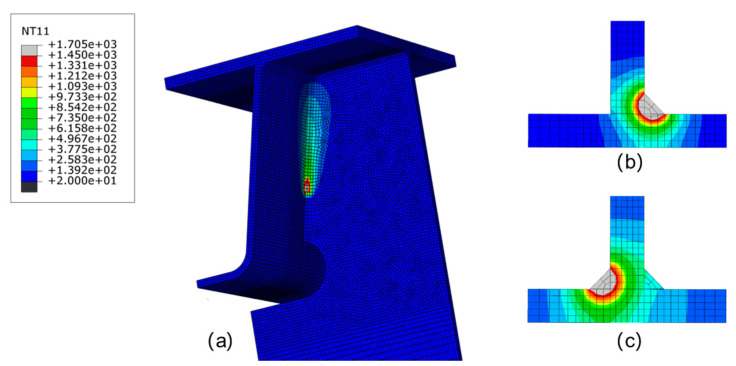
Temperature (unit: °C) contours at the fusion zone and HAZ during welding process: (**a**) top of the fillet (**b**) first side of the fillet, and (**c**) second side of the fillet.

The characteristics of the weld pool can reflect the quality of welding and the range of HAZ. Biswas et al. [42] developed a numerical elastoplastic thermo-mechanical model for predicting the thermal history and the distortion of double-sided fillet joints during submerged arc welding. Figure 7 shows the simulated temperature distribution in the fusion region during the welding process. The simulated isothermal profile at the fusion zone and the heat-affected zone matches closely with test one [42]. When the heat source applied along the first fillet weld (at *t* = 1076.58 s), the temperature field of the joint is displayed in Figure 7a. The area where a temperature is higher than 1450 °C is defined as the fusion zone, and the shape of the melt pools at the first and second welds are illustrated in Figure 7b,c, respectively. The findings of Biswas‘ experiments on the etched section of a double-sided fillet are in good agreement with the results of current study, verifying the accuracy of the established finite element model.

## 4. Experimental Work

**Figure 8:**
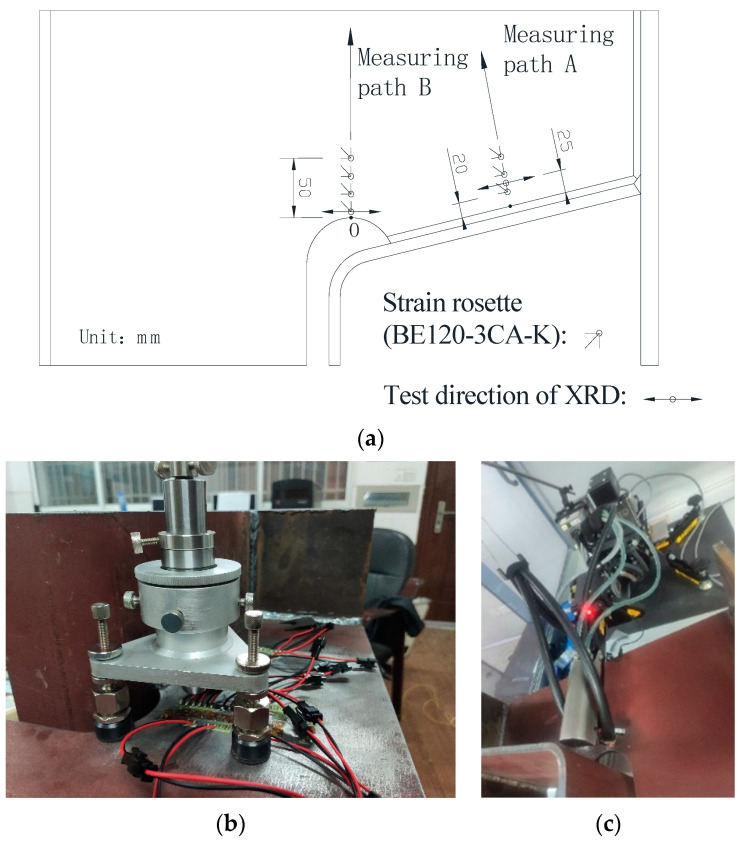
Residual stress measurement: (**a**) measuring points arrangement; (**b**) XRD method, and (**c**) hole-drilling (HD) method.

To verify the numerical simulations using the proposed heat source model, an experimental specimen was fabricated by cutting and welding according to Chinese standards GB/50017-2017 [43]. The experimental specimen was identical to the numerical one, and the working parameters like cutting and welding were set the same as Table 1 and Table 2. Both hole-drilling and x-ray diffraction methods were used to measure the residual stress. The arrangement of the measuring points is presented in Figure 8a.

The hole-drilling (HD) method was used to measure the residual stress in accordance with ASTM E837-08 standards [44], as shown in Figure 8b. The arrangement of the measuring points along Path B near the diaphragm cutout and along Path A around the middle of welding joint is shown in Figure 8a. The first measuring point along path A is 2 mm away from the weld edge, and the distance between the first measuring point along Path B and the cutting free line is 0.5 mm. The spacing between each measuring point is 15 mm. The surface near the measuring points was polished. After thorough cleaning and degreasing with acetone solvent, rosette strain gauges (BE120-3CA-K, produced by NANJING HOPE TECHCO., Ltd., Nanjing, China) were affixed at the center of measurement points. The diameter and depth of the drilled holes are 1.5 mm and 2 mm, respectively. Based on previous experimental practice, the formula for calculating the residual stress are recommended by Wang et al. [45] and all of the parameters in the formulas were measured and provided by NANJING HOPE TECHCO., Ltd.

Nominal stress approach and hot-spot stress approach are often used in the fatigue life prediction of the structures [46]. The HD method is often used to measure the residual stress of steel structures, and the measured values used to predict fatigue life are generally considered reliable [30,45]. However, the HD method is not recommended in the existing codes for higher residual stress areas [43,47]. For proving and complementing the HD method, two representative regions were measured by X ray diffraction (XRD) instrument, which was made by PROTO Manufacturing Ltd. of Canada. Since the depth of the x-ray penetrating material is only from a few microns to several micrometers, the measuring points were initially electropolished using saturated sodium chloride (NaCl) solution. When the base metal at the measurement area was electropolished to be adequately smooth and even, the residual stress at the center of the polished circular area was measured, as shown in Figure 8c. Due to the limited space between the probe and U rib, the measuring points are 0.5 and 25 mm away from the cutting line and weld edge, respectively. The x-ray diffraction method strictly complies with EU standard EN 15305-2008 [48], and the residual stress was directly obtained through the software Protoxrdwin2.0 (PROTO Manufacturing Ltd., Oldcastle, ON, Canada).

## 5. Results and Discussions

### 5.1. Stress and Temperature Time History

The temperature-time history curve can directly reflect the thermal and physical status in the U rib-diaphragm joint of OSDs during the whole manufacture process. In addition, the local stress state of each component of the joint was evaluated by von Mises yield criteria. In order to understand the thermal-mechanical coupling during the whole assembling process, the real-time temperature and stress field were measured at measuring points A and B, as indicated in Figure 1b. And the area that had a maximum temperature above 727 °C is considered to be the HAZ.

**Figure 9:**
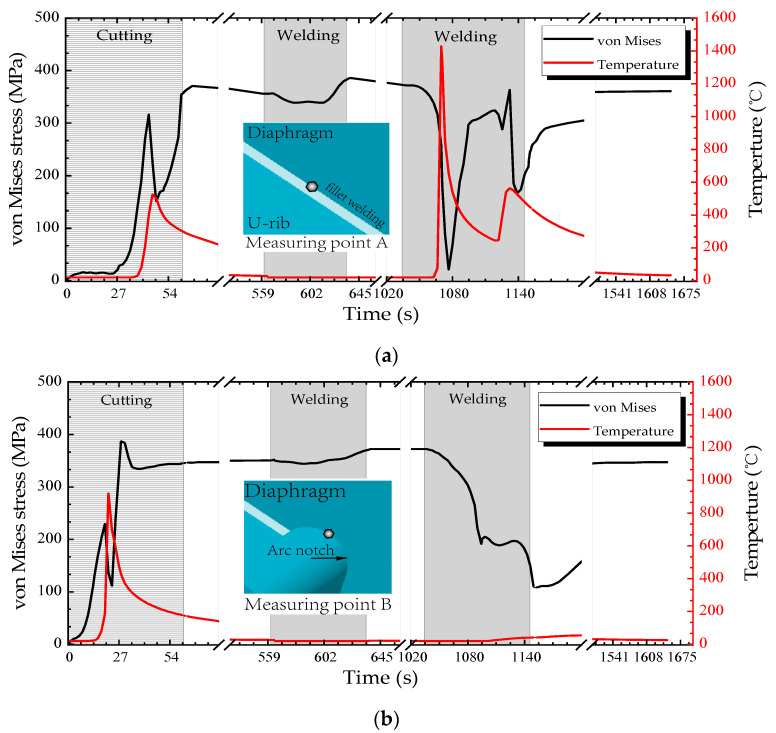
Real-time von Mises stress and temperature: (**a**) measuring point A; and (**b**) measuring point B.

Figure 9a,b show the variations of von Mises stress and temperature with processing time at the measuring points A and B, respectively. First, during the cutting process, the maximum temperature at point B, which is on the cutting line, is 923.021 °C (*t* = 21.39 s), while the maximum temperature at point A, which is far from the cutting line, is 496.45 °C (*t* = 48.87 s). The point B is at the edge of the cutting line, while point A is 6.5 mm from the cutting line. Thus the width of HAZ is limited during the cutting process. So the cutting process mainly changes the material properties at the cutting line, but it is not enough to cause the diaphragm fatigue crack near the weld fillet.

Second, during the process of welding the diaphragm to the deck, the temperature at the points A and B are lower than that during the cutting process. Although the residual stress in the joint of the diaphragm-decks introduced by welding during this process may result in changes of the internal forces at the diaphragm-rib joints, the effects are not obvious. It is recommended to simplify the welding process when studying the residual stress near the cutout.

Finally, during the U rib-diaphragm welding process, the maximum temperature at point A is 1452.79 °C, which reaches the melting point. Thus, the stress variation at point A is mainly caused by flame cutting and U rib-diaphragm welding. Since there is always an obvious distance between the weld fillet and the diaphragm cutout Point B, the temperature at Point B does not change significantly and it is kept at a lower level. Different from stress variation at point A, the stress change at point B is mainly caused by cutting because the temperature effect cannot change the material properties at point B during the U rib-diaphragm welding process. Therefore, the welding of the diaphragm to the deck has little effect on the residual stress near the diaphragm cutout, and the residual stress in the diaphragm cutout is mainly caused by the cutting process.

### 5.2. Residual Stress Distribution

**Figure 10:**
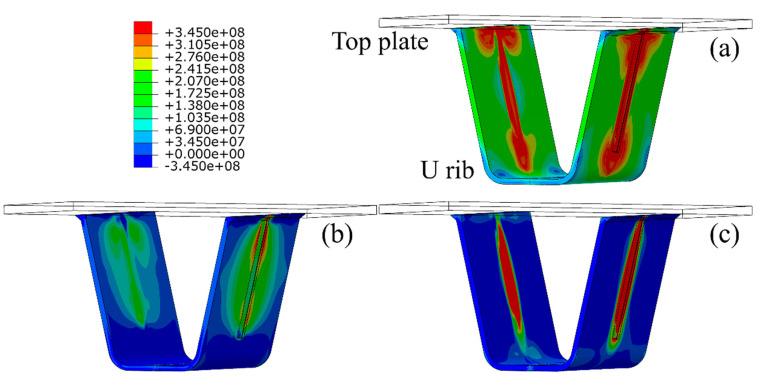
Residual stress distributions around U rib (units: Pa): (**a**) von Mises stress; (**b**) transverse residual stress, and (**c**) longitudinal residual stress.

**Figure 11:**
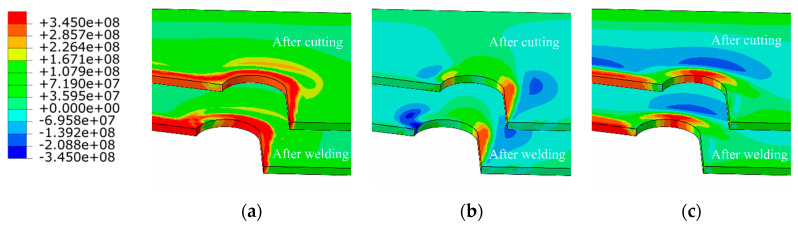
Residual stress distributions in diaphragm cutout (units: Pa): (**a**) von Mises stresses; (**b**) transverse residual stress, and (**c**) longitudinal residual stress.

**Figure 12:**
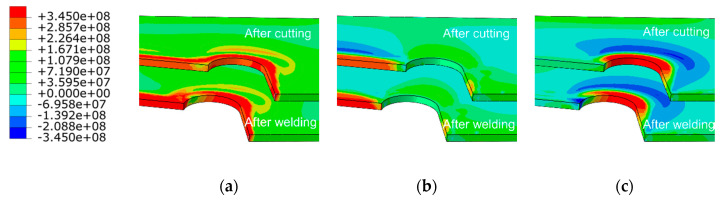
Residual stress distributions in diaphragm cutout (units: Pa): (**a**) von Mises stresses; (**b**) normal residual stress, and (**c**) tangential residual stress.

Since the junction of the diaphragm and U rib in OSDs is prone to fatigue cracking under repeated traffic loading, the residual stress distribution around the U rib was investigated, as shown in Figure 10, in which the left and right panels are the residual stress distribution of the inner (near the diaphragm) and outer (near the opposite side) sides of the U rib, respectively. In Figure 10, the direction parallel to the weld direction is defined as the longitudinal direction, while perpendicular to weld direction and parallel to the U rib direction are defined as the transverse direction. In order to facilitate reading and analysis, the cartesian coordinate system defaulted by ABAQUS is used in the present study, and the stress distributions near the cutout in different coordinate systems were compared to ensure the accuracy of the analysis, as illustrated in Figure 11 and Figure 12. The red regions in stress contour where the value is more than the yield strength are denoted as high stress areas. It can be found that the welding process introduces significant residual tensile stresses, that are mainly concentrated near the weld seam. The residual stress range near the diaphragm is larger than that on the opposite side, and the transverse stresses are significantly lower than the longitudinal stresses along the weld seam.

The stress in the mid of the weld seam tends to be stable, and the width of the stress distribution is uniform. The yield area (i.e., the red region in Figure 10a) substantially expands from the diaphragm to the weld tip of the U rib. Notably, at the boundary of the cutout-rib joint, the longitudinal residual tensile stresses are mainly concentrated near the weld fillet of the U rib (Figure 10c). Thus, the complex distribution of residual stress can be regarded as one of the most important causes of fatigue around U ribs in OSDs.

The distribution of residual stress in the diaphragm cutout is of great significance to the fatigue life of the U rib-diaphragm joint in OSDs. The evolution of residual stress (in terms of von Mises, longitudina,l and transversal residual stress) in the diaphragm cutout are depicted in Figure 11, respectively, in which, the longitudinal stress direction is parallel to the weld, while the transverse direction is perpendicular to weld and parallel to the diaphragm. Flame cutting results in a strip-shaped (width of 10 mm) residual stress field near the diaphragmn cutout. The residual tensile stress is mainly distributed along the cutting line, and concentrated in the middle of the arc-shaped section of the diaphragm cutout.

After welding, the von Mises stress in the joint of the diaphragm cutout and U rib drops below the yield strength U rib, indicating an improvement in the stress concentration in this region. The welding process causes a large longitudinal compressive stress of 278 MPa at the junction of the diaphragm cutout and the welds (dark-blue area), as shown in the Figure 11b. However, the variation of the transverse residual stress near the cutout is not obvious before and after welding, because of the influence of longitudinal constraints by the welding joint and a relative far distance from the heat-affected zone. The concentration of residual stress in the diaphragm cutout, especially for the longitudinal tensile stress near the the middle of the arc-shape section, is considered to be an important reason for fatigue cracking.

The cutting residual stresses are often distributed along the cutting line, and the residual stresses along tangent direction are much higher than the others‘ direction. For analyzing the residual stress distributions in the diaphragm cutout, the rectangular coordinate system was specially replaced by the cylindrical coordinate system. The residual stress distributions in the cylindrical coordinate axis are shown Figure 12, similar to those described in Figure 11. The residual stress along the straight line is still distributed along the cutting seam. Furthermore, the peak value of residual stress in tangential direction reaches 377.8 Mpa, which is close to the value of the Cartesian coordinate system established above. Consequently, the analysis of the residual stress distributions along Path B in the default coordinate system is considered to be sufficiently accurate.

**Figure 13:**
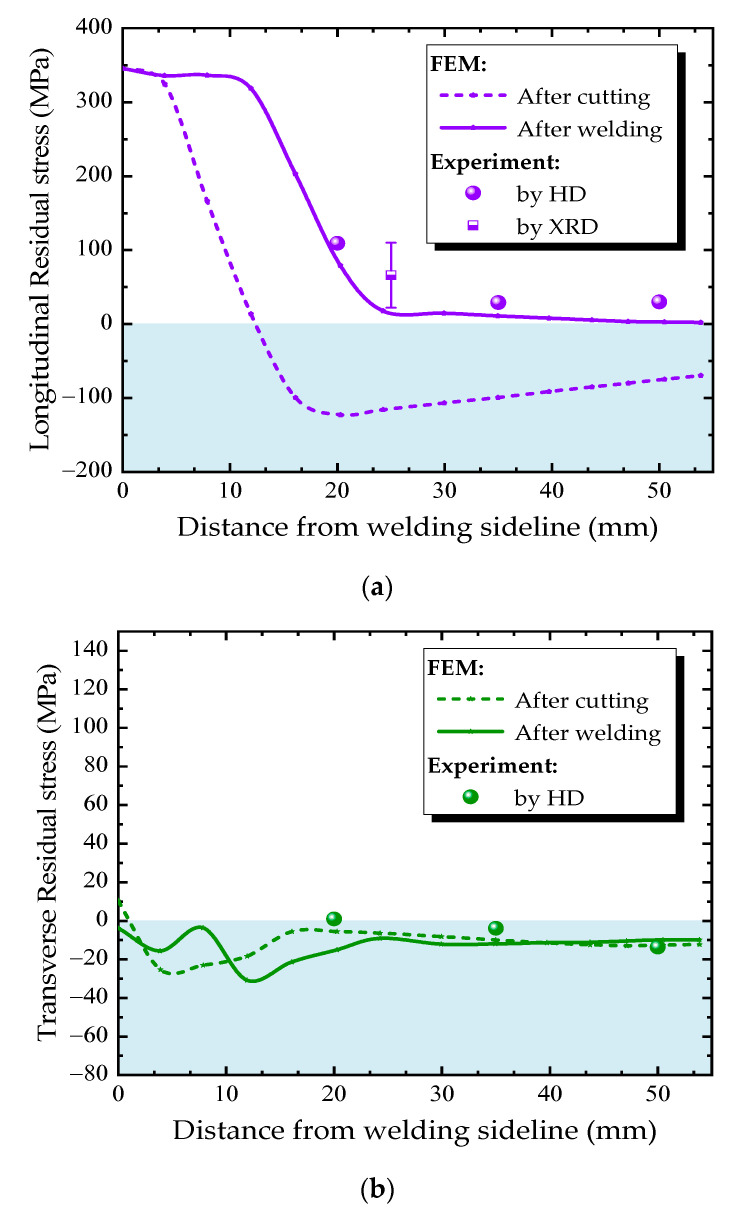
Residual stress distributions along Path A: (**a**) longitudianal direction, (**b**) transverse direction.

**Figure 14:**
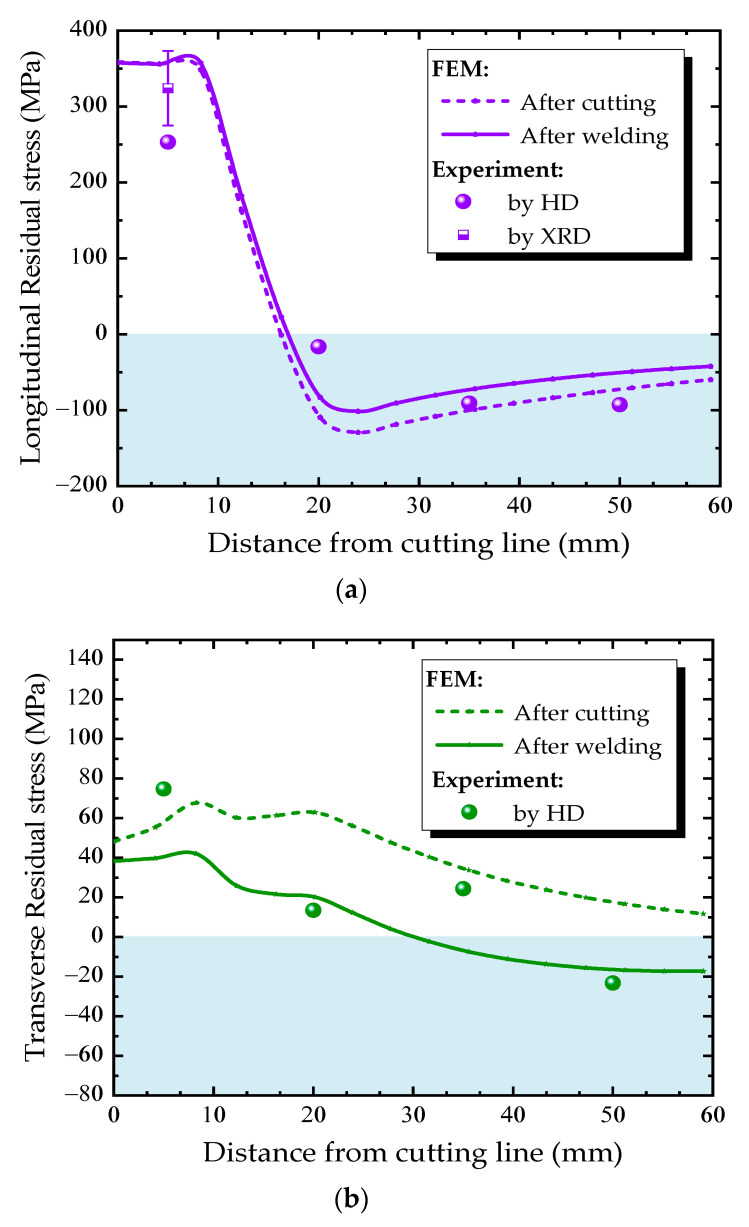
Residual stress distributions along Path B: (**a**) longitudinal direction, (**b**) transverse direction.

Figure 13 and Figure 14 compare the residual stress distributions along the measuring paths A and B (Figure 8a) at the diaphragm obtained from finite element simulation and experimental measurement. The residual stress varies greatly along both transverse and longitudinal directions. The trend of simulated residual stress is consistent with the measured one. The measured values using the HD method are in good agreement with the simulated one in the region where the residual stresses are small. However, in the high residual stress region, the HD method underestimates the residual stress, while the x-ray diffraction method can accurately reflect the actual residual stress.

The residual stress distribution along the measured Path A is shown in Figure 13. The residual stress caused by the cutting process is mainly distributed along the longitudinal direction, and the value is larger near the cutting line, but decreases gradually with the distance away from the cutting line. The high tensile stress zone caused by cutting is narrow—the width of high stress area is only 5 mm. Then, the welding process of the diaphragm cutout connecting to U-rib extends the longitudinal tensile stress from the weld fillet toward the center of the diaphragm. The welding process increases the width of the high stress zone to nearly threefold (from 5 mm to 15 mm), but the peak value of residual stress does not change significantly. The cutting and welding residual stresses are all transformed into compressive stresses which are away from the cutting line. During the whole fabrication process, the transverse tensile stress was maintained at low level, and did not change obviously. Thus, longitudinal residual stresses in U rib-diaphragm joints in OSDs should be paid more attention.

The residual stress distribution along the measured Path B near the diaphragm cutout is shown in Figure 14. There is a region with large residual stress near the cutting line, the maximum value approaches to 375 MPa at about 10 mm from the cutting line. In addition, the residual stress decreases with the distance from the cutting line, even changes from tensile stress to compressive stress, and reaches maximum compressive stress at approximately 23 mm from the weld fillet, then decreases gradually. Form Figure 9b, it can be seen that the temperatures at Path B remain below 100 °C during the welding process, this is the reason why the variation of residual stress is not obvious.

### 5.3. Effects of Cutting and Welding Speed

Generally, fabrication procedure directly affects the residual stress distribution of U rib-diaphragm joint in OSDs, such as the movement speed of the heat source induced by cutting or welding determining the heat input per second. Therefore, the effects of cutting and welding speed on the residual stress in diaphragm cutout were investigated using the above-validated numerical simulation method in this section.

**Figure 15:**
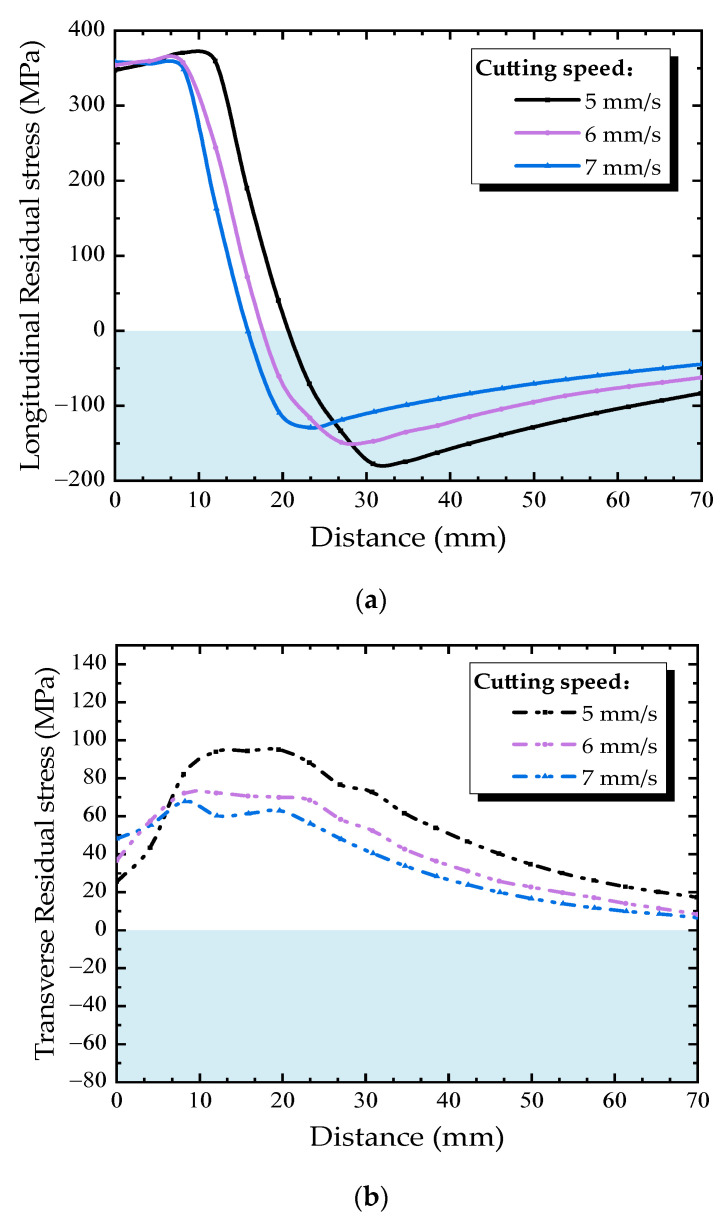
Residual stress distributions along Path B under different cutting speeds: (**a**) longitudinal direction, (**b**) transverse direction.

The effect of cutting speed on the residual stress distribution in diaphragm cutout was studied under cutting speeds of 5, 6, and 7 mm/s and a constant welding speed of 4 mm/s. The residual stress distribution along Path B under different cutting speeds is shown in Figure 15. As for the longitudinal residual stress, with the increase of cutting speed, the peak tensile and compressive stress decrease slightly, also the width of the high stress zone is reduced. Thus, during the thermal cutting process, a higher cutting speed is good in bringing down the width of the high residual stress zone near the free edge of diaphragm cutout. As the cutting speed increases, the transverse tensile stress increases slightly along Path B. The peak tensile stress occurs at about 15 mm away from the cutting line, then transverse tensile stress decreases with the distance away from the cutting line, besides, the faster cutting speed, the smaller transverse tensile stress at the location where the distance is more than 10 mm from cutting line.

**Figure 16:**
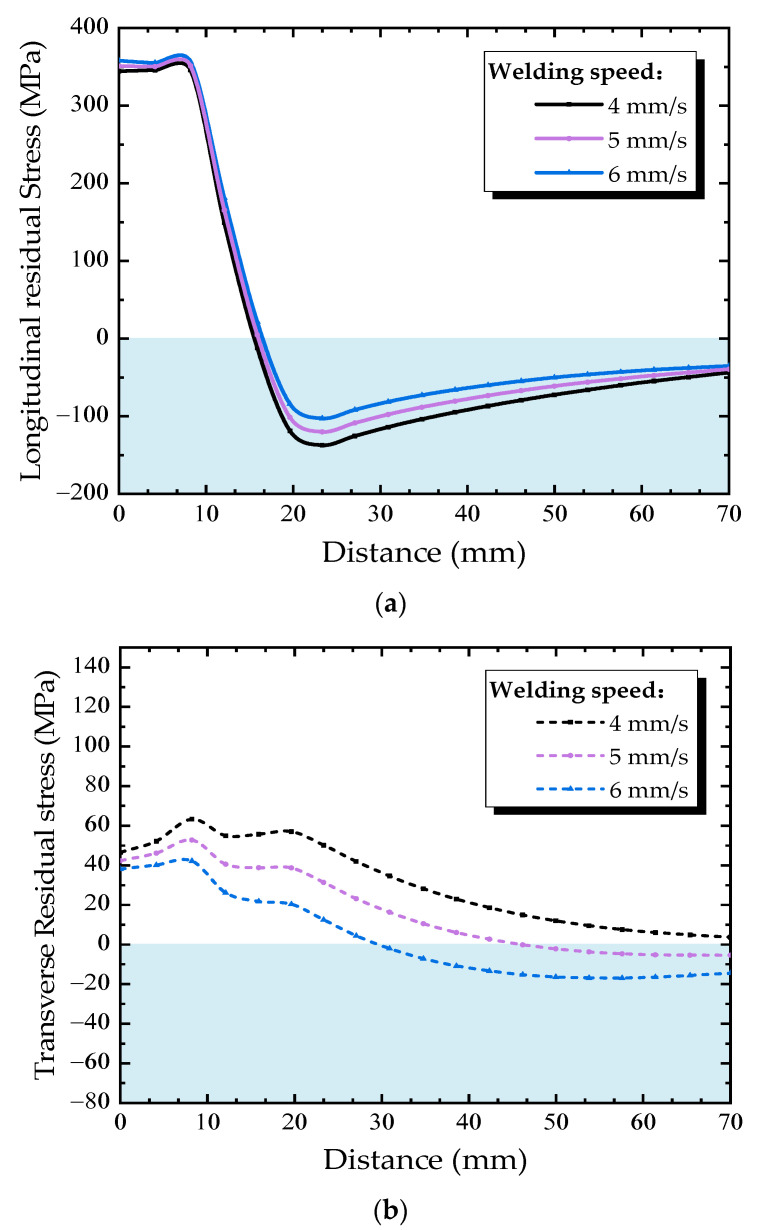
Residual stress distributions along Path B under different welding speeds: (**a**) longitudianal direction, (**b**) transverse direction.

In order to understand the effect of welding speed on the residual stress in the diaphragm cutout, welding speeds of 4, 5, and 6 mm/s and a constant cutting speed of 7 mm/s were selected. Figure 16 shows the residual stress distribution along Path B under different welding speed. The longitudinal residual stress almost shows the same variation trend under different welding speed, as shown in Figure 15a, with the increasing of welding speed, the longitudinal tensile residual stress slightly increases, but the compressive residual stress slightly decreases. The main reason is that the heat source caused by welding is far away from the diaphragm cutout, hence there is no essential change in the residual stress distribution in diaphragm cutout due to the welding process.

Reducing welding speed can increase the heat input per second during the welding process. The HAZ widens under the effect of the welding heat sources, causing the welding residual stress curves to move toward the center of the diaphragm. Each of the points in Path B are more affected than before, thus result in reducing longitudinal residual stresses and raising transverse residual stresses along Path B. Therefore, it is helpful to reduce the residual tensile stress at the diaphragm cutout using a slow welding speed during welding processes of U rib-diaphragm joints in OSDs.

## 6. Conclusions

In the present study, the residual stress field in the joints of the U-rib and transverse diaphragm subjected to thermal cutting and welding is analyzed by numerical simulation as well as x-ray diffraction and hole-drilling tests. The following conclusions can be drawn.

(1) The residual stress around the diaphragm cutouts is mainly caused by the flame cutting process and distributes along longitudinal direction of the cutting line. The established heat source model caused by cutting can accurately describe the temperature distribution along the cutting line. The high residual stress region with a width of about 10 mm is responsible for the fatigue cracking of the diaphragm cutouts.
(2) Near the welding area, the residual stress is mainly introduced by the welding. The longitudinal residual tensile stress (along the weld direction) exists in the weld joints between U-rib and diaphragm, and the peak residual stress even exceeds the yield strength. Moreover, the residual stress concentrates at the boundary of the weld fillets, which should be paid more attention.
(3) The numerical simulation of residual stress distribution during cutting and welding process was validated by experimental measurements using both x-ray diffraction and HD methods. The longitudinal residual stress was found to be higher than the transversal one. In the high residual stress zone, the HD method underestimates the residual stress, while the x-ray diffraction method can accurately predict the actual residual stress.
(4) The width of the high stress zone near the cutting line decreases with cutting speed. The residual stresses in diaphragm cutouts increases with welding speed, but the width of the high stress zone does not change significantly. Hence, choosing a fast cutting speed and a slow welding speed during fabrication processes can reduce the residual stresses and its concentration area at diaphragm cutout, which is beneficial for the fatigue performance of U rib-diaphragm joints in OSDs.

## Figures and Tables

**Table 1:** Parameters used for the cutting heat source model.

Parameters	$Q_{1}/J\cdot m^{-3}$	$Q_{2}/J\cdot m^{-3}$	$R_{1}/mm$	$R_{2}/mm$	H/mm	$V/mm\cdot s^{-1}$
Value	$3.005\times 10^{4}$	$3.525\times 10^{10}$	4.1	1	10	7

**Table 2:** Parameters used for the welding heat source model.

Parameters	b/mm	**c/mm**	$a_{f}/mm$	$a_{r}/mm$	$V/mm\cdot s^{-1}$	I/A	U/V
Value	9	8	6	14	4	250	25
